# Cyanase-independent utilization of cyanate as a nitrogen source in ascomycete yeasts

**DOI:** 10.1007/s11274-018-2579-4

**Published:** 2018-12-13

**Authors:** Tomas Linder

**Affiliations:** Department of Molecular Sciences, Swedish University of Agricultural Sciences, Box 7015, 750 07 Uppsala, Sweden

**Keywords:** Cyanase, Cyanate, Metabolism, Phenotype, Yeast

## Abstract

The occurrence of putative cyanases (EC 4.2.1.104) in the genomes of yeasts belonging to the ascomycete sub-phyla Saccharomycotina (budding yeasts) and Taphrinomycotina (fission yeasts) was investigated. Predicted gene products displaying significant sequence similarity to previously characterized cyanases were identified in the genomes of the budding yeast *Lipomyces starkeyi* and the fission yeasts *Protomyces lactucaedebilis, Saitoella complicata* and *Taphrina deformans. Li. starkeyi* and *Sai. complicata* were further tested for their ability to utilize cyanate as a nitrogen source. However, neither species displayed significant growth when cyanate was provided as the sole nitrogen source. Cyanate utilization assays of 15 yeast species whose genomes lack detectable cyanase homologs unexpectedly resulted in consistently strong growth in six species as well as variable growth in an additional three species. The present study represents the first known report of cyanase-independent utilization of cyanate as a nitrogen source in ascomycete yeasts. Implications of cyanate utilization for the ecological niches occupied by ascomycete yeasts are discussed.

## Introduction

Cyanate (OCN^−^) is a naturally occurring toxic compound that can be produced by a number of abiotic processes including oxidation of cyanide (CN^−^) or the spontaneous decomposition of simple nitrogenous biomolecules such as urea or carbamoyl phosphate. Cyanate has been shown to inhibit a number of essential enzymes (Anderson et al. 1973; Jain and Kassner 1984; Peng et al. 1993) and can also lead to non-specific carbamoylation of proteins (Kraus et al. 1994). Sea water has been shown to contain cyanate in the 1–40 nM range (Widner et al. 2013) while substantially higher concentrations can be found in treated mine effluents (Sancho et al. 2005). Many plants produce cyanide in response to herbivores and phytopathogens, which subsequently becomes oxidized into cyanate. Although cyanate is less toxic than cyanide, the toxicity of cyanate is still sufficient to create a selection pressure for the development of cyanate detoxification mechanisms in organisms that target cyanogenic plants.

The enzyme cyanase (EC 4.2.1.104; also known as cyanate hydratase or cyanate lyase) degrades cyanate into CO_2_ and ammonia using bicarbonate (HCO_3_^−^) as a co-substrate according to the reaction:

$${\text{OC}}{{\text{N}}^ - }+{\text{ HC}}{{\text{O}}_3}^{ - }+{\text{ }}2{\text{ }}{{\text{H}}^+} \to 2{\text{ C}}{{\text{O}}_2}+{\text{ N}}{{\text{H}}_3}$$

The cyanase gene was first cloned from the bacterium *Escherichia coli* (Sung et al. 1987) and has since been found in numerous bacteria (Espie et al. 2007; Luque-Almagro et al. 2008), plants (Qian et al. 2011) and fungi (Elleuche and Pöggeler 2008). There is even one reported case of an ancient horizontal transfer event of a functional cyanase into the genomes of phytophagous spider mites belonging to the family Tetranychidae (Wybouw et al. 2012). Cyanase also enables bacteria to utilize cyanate as a nitrogen source for growth (Kunz and Nagappan 1989; Sung et al. 1987).

Cyanate metabolism has not been studied among ascomycete yeasts belonging to the sub-phyla Saccharomycotina (budding yeasts) and Taphrinomycotina (fission yeasts). Cyanase homologs have previously been reported as being absent in the genomes of budding yeasts (Elleuche and Pöggeler 2008; Elmore et al. 2015) while the utilization of cyanate as a nitrogen source has not been investigated in either sub-phylum. However, the number of sequenced yeast genomes has increased rapidly in recent years as large-scale DNA sequencing has become cheaper. The number of budding yeast species with sequenced genomes exceed 300 at the time of writing. The current number of described fission yeast species remains low but a high proportion of these species have sequenced genomes. The present study therefore sought to revisit the occurrence of cyanase homologs among ascomycete yeast genomes and whether the possession of a cyanase homolog correlated with the ability to utilize cyanate as a nitrogen source.

## Materials and methods

### Protein sequence analysis

Potential yeast homologs of the *E. coli* cyanase (NCBI GenBank protein accession number AAA23629) were identified through BLASTP searches against the NCBI GenBank nr and reference sequence protein databases using an expect (*E*) value threshold of 10^−6^ and a taxonomic filter for either sub-phylum Saccharomycotina (taxid: 147537) or Taphrinomycotina (taxid: 451866). Yeast draft genomes currently lacking gene annotations were queried through a TBLASTN search with the *E. coli* cyanase protein sequence against the NCBI GenBank whole genome shotgun (wgs) DNA database using an *E* value threshold of 10^−6^ and a taxonomic filter as described above. The genomes of two yeast species that were used in subsequent nitrogen utilization assays were queried using customized BLAST servers. The protein complement of the *Lachancea kluyveri* genome was investigated for cyanase homologs using the GRYC BLASTP server (http://igenolevures.org/) with an *E* value threshold of 10^−6^. The protein complement of the *Sugiyamaella americana* genome was investigated for cyanase homologs using the Joint Genome Institute *Su. americana* BLASTP server (https://genome.jgi.doe.gov/pages/blast-query.jsf?db=Sugame1) with an *E* value threshold of 10^−6^.

Putative cyanase protein sequences were aligned using MAFFT (Katoh et al. 2005; https://mafft.cbrc.jp/alignment/server/) using the G-INS-i setting and formatted in BoxShade v. 3.21 (https://embnet.vital-it.ch/software/BOX_form.html). Yeast homologs of the *Sac. cerevisiae* carbonic anhydrase (NCBI GenBank protein accession number NP_014362) were identified through BLASTP searches against the NCBI GenBank nr and reference sequence protein databases using an *E* value threshold of 10^−6^ and a taxonomic filter for the corresponding species.

### Yeast growth assays

The yeast strains used in this study are listed in Table 1. *Schizosaccharomyces pombe* strain Leu972 was a kind gift from Dr. Pernilla Bjerling (Uppsala University, Sweden). All other strains were purchased from the Westerdijk Fungal Biodiversity Institute (Utrecht, the Netherlands). Sodium glutamate and sodium cyanate was purchased from Sigma Aldrich. Yeast growth assays in chemically defined minimal medium with individual nitrogen sources has been described previously (Linder 2018). Briefly, a reduced sulfur/nitrogen-limited glucose medium (RSNLD) was used for assaying growth on either l-glutamate or cyanate. RSNLD medium is composed of 1.7 g/l Difco yeast nitrogen base without amino acids or ammonium sulfate (Becton, Dickinson and Company) and 20 g/l glucose. Prior to growth assays, individual yeast strains were pre-cultured overnight in 3 ml minimal glucose medium consisting of 6.7 g/l Difco yeast nitrogen base without amino acids (Becton, Dickinson and Company) and 20 g/l glucose. Pre-cultures were washed twice in RSNLD and then re-suspended in 2.97 ml RSNLD to a final OD_600_ of 0.005 in a 50 ml tube. Sodium l-glutamate or sodium cyanate was added as 30 μl of a 1 M stock solution to a final sample concentration of 10 mM. A non-supplemented sample with 30 μl deionized water was used as a control. Chloramphenicol (final concentration 15 mg/l) was included to prevent bacterial contamination. Growth assays were conducted at 30 °C in a rotary shaker set to 200 rpm with OD_600_ measurements 6, 12 and 18 days after inoculation. All growth assays were performed in triplicate.

**Table 1:** Yeast strains used in this study

Species name	Strain number	Family	Clade^a^
Budding yeasts (sub-phylum Saccharomycotina)			
* Cyberlindnera jadinii*	CBS 5609	Phaffomycetaceae	Leu1
* Kluyveromyces marxianus*	CBS 6556	Saccharomycetaceae	Leu1
* Komagataella pastoris*	CBS 704	Saccharomycetales incertae sedis	Leu2
* Kuraishia capsulata*	CBS 1993	Saccharomycetales incertae sedis	Leu2
* Lachancea kluyveri*	CBS 3082	Saccharomycetaceae	Leu1
* Lipomyces starkeyi*	CBS 1807	Lipomycetaceae	Leu0
* Ogataea parapolymorpha*	CBS 11895	Pichiaceae	Leu2
* Pachysolen tannophilus*	CBS 4044	Saccharomycetales incertae sedis	Ala
* Scheffersomyces stipitis*	CBS 6054	Debaryomycetaceae	Ser1
* Spathaspora passalidarum*	CBS 10155	Debaryomycetaceae	Ser1
* Starmerella bombicola*	CBS 6009	Trichomonascaceae	Leu0
* Sugiyamaella americana*	CBS 10352	Trichomonascaceae	Leu0
* Tortispora caseinolytica*	CBS 7881	Trigonopsidaceae	Leu0
* Yamadazyma tenuis*	CBS 615	Debaryomycetaceae	Ser1
* Yarrowia lipolytica*	CBS 7504	Dipodascaceae	Leu0
Fission yeasts (sub-phylum Taphrinomycotina)			
* Saitoella complicata*	CBS 7301	Protomycetaceae	–
* Schizosaccharomyces pombe*	Leu972	Schizosaccharomycetaceae	–

^a^See Krassowski et al. (2018)

## Results

The occurrence of cyanase homologs in the genomes of ascomycete yeasts belonging to either the budding yeasts or the fission yeasts was investigated using the substantial amount of genomic sequences currently available. The *E. coli* cyanase protein sequence was used to query both the protein and DNA databases (the latter translated into protein in all six reading frames) at GenBank for potential homologs among budding and fission yeasts.

**Fig. 1:**
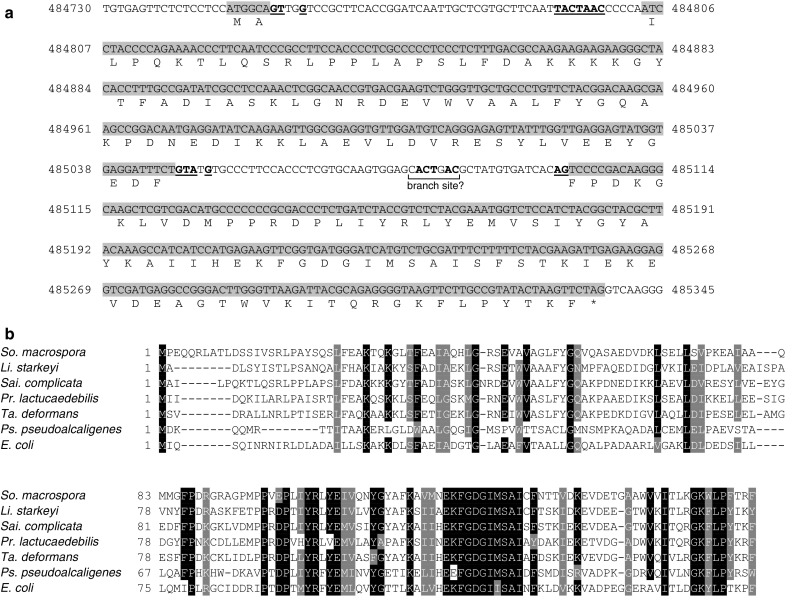
Cyanase homologs among ascomycete yeasts. **a** A revised gene prediction for a putative *Sai. complicata* cyanase-encoding gene. Coding sequences are shaded grey with the corresponding protein sequence shown underneath. Canonical splice motifs are indicated in bold underlined font. Sequence coordinates for *Sai. complicata* unplaced genomic scaffold SAICOscaffold_6 are shown (GenBank DNA accession NW_017566997). **b** Multiple sequence alignment of cyanase-like protein sequences of *Sordaria macrospora* (filamentous ascomycete fungus, GenBank protein accession CAO79555), *Li. starkeyi* (budding yeast, GenBank protein accession ODQ76668), *Sai. complicata* (see previous figure), *Pr. lactucaedebilis* (fission yeast, GenBank protein accession ORY84881), *Ta. deformans* (fission yeast, GenBank protein accession CCG81385), *Pseudomonas pseudoalcaligenes* (gamma-proteobacterium, GenBank protein accession ABO37842) and *E. coli* (gamma-proteobacterium, GenBank protein accession AAA23629). 80% of residues at each aligned position must be either identical (black) or similar (grey) for shading

Putative cyanase homologs were identified in the genomes of the fission yeasts *Protomyces lactucaedebilis, Saitoella complicata* and *Taphrina deformans* but not in the genome of the common fission yeast model system *Schi. pombe*. A putative and hitherto unreported cyanase gene was also identified the related multicellular archiascomycete *Neolecta irregularis* (Nguyen et al. 2017) through protein similarity searches against translated whole-genome shotgun scaffolds (not shown). *Lipomyces starkeyi* was the only budding yeast whose genome contained a putative cyanase homolog. Yeast cyanase homologs were of similar size (158 amino acids) to previously described cyanases with the exception of the predicted *Sai. complicata* cyanase homolog, which was significantly larger at 1066 amino acids (GenBank protein accession XP_019025654). The cyanase-like sequence was located to the N-terminus of the predicted *Sai. complicata* protein, which raised the possibility that the apparent fusion protein was an artifact of the gene prediction algorithm (Riley et al. 2016). A revised gene prediction for the putative *Sai. complicata* cyanase-encoding gene was carried out manually using the *Pr. lactucaedebilis* and *Ta. deformans* protein sequences to identify conserved protein sequences in translated genomic DNA sequence. A possible gene model was arrived at, where the putative cyanase coding sequence was spread over three exons (Fig. 1a). The first coding exon contained only the first two codons followed by a predicted 51-bp intron containing both a canonical 5′ donor splice site as well as a canonical branch site (5′ TACTAAC 3′) but lacked a canonical 3′ acceptor splice site (Kupfer et al. 2004). The second and third coding exons were separated by a predicted 54-bp intron containing both canonical 5′ donor and 3′ acceptor splice sites while the predicted branch site (5′ CACTGAC 3′) deviated from the ideal sequence motif by two nucleotides. The resulting protein sequence consisted of 161 amino acids and aligned better with other cyanases (Fig. 1b), which suggested that this gene model better reflected the true *Sai. complicata* cyanase transcript.

All described cyanases require bicarbonate as a co-substrate for function (Johnson and Anderson 1987). Intracellular bicarbonate is produced from CO_2_ and water through the action the enzyme carbonic andydrase (EC 4.2.1.1; Amoroso et al. 2005). In filamentous ascomycetes of the sub-phylum Pezizomycotina, the cyanase gene often co-localizes with carbonic anhydrase in a cluster called CCA (Elmore et al. 2015). Co-localization of the genes encoding cyanase and carbonic anhydrase in the yeasts *Li. starkeyi, Pr. lactucaedebilis, Sai. complicata* and *Ta. deformans* was investigated by identifying the corresponding genomic locations for both genes. The *Sac. cerevisiae* carbonic anhydrase Nce103 (Amoroso et al. 2005) was used to query GenBank protein databases for the corresponding orthologs. All four yeasts possessed a single protein that was homologous to the *Sac. cerevisiae* carbonic anhydrase. However, all four orthologous carbonic anhydrase genes consistently mapped to separate genomic scaffolds than the corresponding putative cyanase-encoding genes (data not shown).

Bacteria have previously been shown to use cyanase in the assimilation of cyanate as a source of nitrogen (Kunz and Nagappan 1989; Sung et al. 1987) while the utilization of cyanate as a nitrogen source has not been previously reported in fungi. The two cyanase-containing yeasts *Li. starkeyi* and *Sai. complicata* were therefore selected for further studies of cyanate utilization. An additional 15 species of yeast (14 budding yeasts and one fission yeast; Table 1), whose genomes lacked detectable cyanase homologs, were included for comparison. The species of budding yeast lacking putative cyanases were selected based on taxonomic distribution and an effort was made to sample a representative cross-section of the sub-phylum Saccharomycotina (Krassowski et al. 2018).

**Fig. 2:**
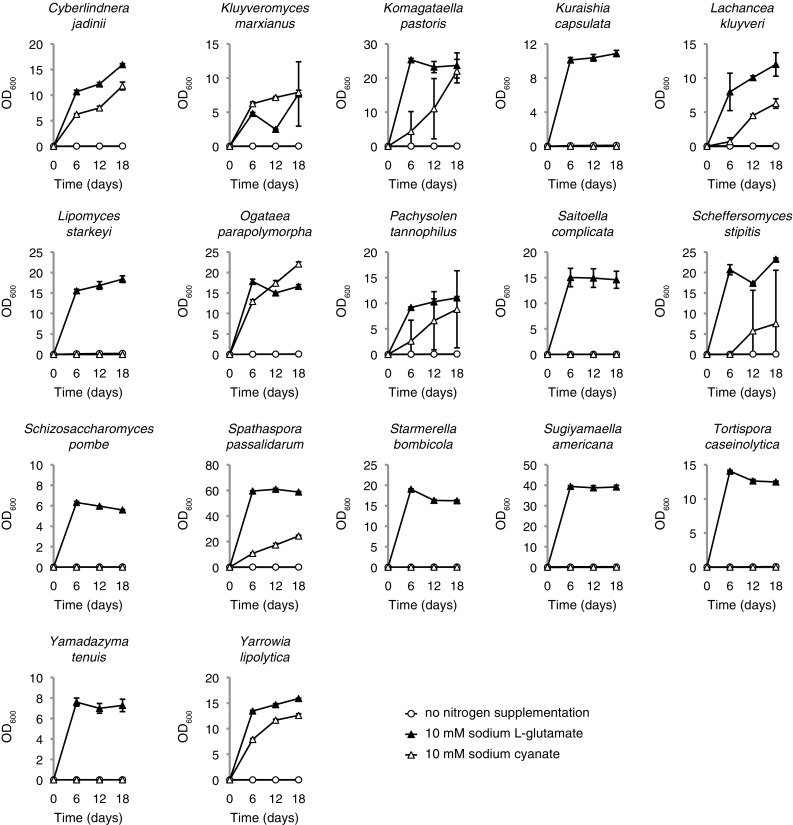
The utilization of cyanate as a nitrogen source in ascomycete yeasts. Yeast strains were cultured in 3 ml chemically defined minimal medium (Linder 2018) containing either 10 mM of the indicated nitrogen source or no nitrogen supplementation (initial OD_600_ 0.005). Samples were incubated in a shaker set at 30 °C, 200 rev/min, and OD_600_ was measured after 6, 12 and 18 days. Growth assays were performed in triplicate with error bars indicating one standard deviation

*Li. starkeyi* and *Sai. complicata* unexpectedly failed to display detectable growth in chemically defined minimal media when the sodium salt of cyanate was provided as the sole source of nitrogen (Fig. 2). Both species displayed consistently strong growth when sodium l-glutamate was provided as the sole nitrogen source, which demonstrated that there was no other limiting nutrient beyond nitrogen. Another unexpected observation was the consistently strong growth that was observed in the yeasts *Cyberlindnera jadinii, Kluyveromyces marxianus, La. kluyveri, Ogataea parapolymorpha, Spathaspora passalidarum* and *Yarrowia lipolytica* when cyanate was provided as the sole source of nitrogen. In addition highly variable growth on cyanate was observed in *Komagataella pastoris, Pachysolen tannophilus* and *Scheffersomyces stipitis* where some biological replicates displayed strong growth while other biological replicates displayed weak or barely detectable growth. No detectable growth could be observed in *Kuraishia capsulata, Schi. pombe, Starmerella bombicola, Su. americana, Tortispora caseinolytica* or *Yamadazyma tenuis* when cyanate was provided as the sole source of nitrogen. All tested species grew well on sodium l-glutamate as sole nitrogen source.

## Discussion

The utilization of cyanate by ascomycete yeasts has not been investigated previously and the present study is the first to report the identification of cyanase homologs in the genomes of the budding yeast *Li. starkeyi* and the three fission yeasts *Pr. lactucaedebilis, Sai. complicata* and *Ta. deformans* (Fig. 1a, b). However, the yeast nitrogen utilization assays described here challenge the current understanding of not only cyanase function but also cyanate utilization by yeasts as well as potentially other microorganisms. The first unexpected observation was that *Li. starkeyi* and *Sai. complicata*, whose genomes contained genes homologous to previously described bacterial and fungal cyanases, failed to show any detectable growth when provided with cyanate as the sole source of nitrogen (Fig. 2). The second unexpected observation was the robust growth displayed in six species whose genomes lacked detectable cyanase homologs. The first question therefore becomes what the roles of the yeast cyanase homologs are if they do not convey the ability to utilize cyanate as a nitrogen source. It should be emphasized that the two fission yeasts *Pr. lactucaedebilis* and *Ta. deformans* were not assayed for cyanate utilization in the present study and therefore it cannot be excluded that cyanase-mediated cyanate utilization occurs in one of both of these species. With regards to *Li. starkeyi* and *Sai. complicata*, one possibility is that the corresponding cyanase-like genes are in fact responsible for cyanate catabolism but only in the context of cyanate detoxification and are therefore not regulated by nitrogen availability. The other possibility is that the resulting gene products in *Li. starkeyi* and *Sai. complicata* target another substrate than cyanate. Both genes remain to be characterized genetically in addition to the biochemical characterization of the resulting proteins.

The second more pressing question is what enzyme or enzymes are required for cyanate utilization in budding yeasts. The six species that displayed consistent growth, displayed cell densities of the same magnitude as the control substrate sodium l-glutamate, which would indicate that an established cyanate catabolic pathway exists under the regulation of nitrogen availability. Three additional species—*Ko. pastoris, Pa. tannophilus* and *Sche. stipitis*, displayed highly variable growth between biological replicates. This notable variation in growth dynamics within single strains of these three species has not been observed previously for other nitrogen sources by the author (Linder 2014). One possibility is that the regulatory circuits for cyanate utilization in these species are not as sensitive to nitrogen availability and therefore only a subset of the cell population will successfully activate the pathway (or pathways) for cyanate utilization. Another possibility is the presence of spontaneous mutants within the yeast population that result in less efficient enzymes or cyanate transporters, which would manifest itself as weaker growth in these samples.

Another open question is how the ability of yeasts to utilize cyanate as nitrogen source correlates with their corresponding ecological niches. Many budding yeasts are associated with phytophagous insects (Blackwell 2017) and it is possible that some endosymbiotic yeasts assist the host in the detoxification of plant-derived cyanate. *Li. starkeyi* is situated at the base of the budding yeast sub-phylum (Riley et al. 2016). If the cyanase gene was initially lost in other budding yeast lineages before insect–yeast associations were established, this could have lead to the appropriation of other metabolic pathways for cyanate catabolism. The eventual identification of this novel cyanate catabolic pathway in budding yeasts should resolve this question.

It is worth noting that the fission yeasts *Pr. lactucaedebilis* and *Ta. deformans* are plant pathogens, which could promote the development of pathways for cyanate detoxification similarly to what has been described in filamentous ascomycetes (Elleuche and Pöggeler 2008; Elmore et al. 2015). Conversely, *Li. starkeyi* and *Sa. complicata* are both associated with soil. If the cyanase homologs in these two species are in fact specific for cyanate (although not for the purpose of nitrogen assimilation), this may reflect a general requirement for cyanate detoxification in soil ecosystems.

On a final note, the ability of yeasts to utilize cyanate as a nitrogen source can be applied in yeast classification. Despite the emergence of high-throughput technologies for the classification of yeasts based on genomic DNA sequences or chemical fingerprints, simple carbon and nitrogen assimilation tests are still used routinely for identification of yeast species (Kurtzman et al. 2011). It also remains to be established how the ability to utilize cyanate as a nitrogen source correlates with taxonomic ranks such as genus or family as well as among different isolates of a single species.
